# What happens in Brazil? A pandemic of misinformation that culminates in an endless disease burden

**DOI:** 10.1590/0037-8682-0713-2020

**Published:** 2020-12-21

**Authors:** Cristina Ribeiro de Barros Cardoso, Ana Paula Morais Fernandes, Isabel Kinney Ferreira de Miranda Santos

**Affiliations:** 1 Universidade de São Paulo, Faculdade de Ciências Farmacêuticas de Ribeirão Preto, Departamento de Análises Clínicas, Toxicológicas e Bromatológicas, Ribeirão Preto, SP, Brasil.; 2 Universidade de São Paulo, Escola de Enfermagem de Ribeirão Preto, Departamento de Enfermagem Geral e Especializada, Ribeirão Preto, SP, Brasil.; 3 Universidade de São Paulo, Faculdade de Medicina de Ribeirão Preto, Departamento de Bioquímica e Imunologia, Ribeirão Preto, SP, Brasil.

Brazil is currently one of the main centers of the coronavirus disease 2019 (COVID-19) pandemic. Despite the low testing rates, recent epidemiological data estimates more than 5.8 million cases and more than 165.000 cumulative deaths in the country; an alarming number. The pandemic follows an uncontrolled rhythm, with insufficient sanitary rules and inadequate orientation for the population, which is not completely aware of the threats of the pandemic. The inefficient official communication, which is not based on scientific evidence, also contributes to the spread of the disease in Brazil. In addition, the different levels of population incomes create clear discrepancies, leading to early access to medical support services for high-net-worth subjects, giving them a definite advantage. Furthermore, the nutritional status of the population is also highly influenced by education and purchasing power; therefore, obesity is an important comorbidity predominant among underprivileged Brazilians^1^.

Apart from these discrepancies, Brazil is a fertile field for misinformation that hinders adequate measures taken to mitigate COVID-19. For example, chloroquine/hydroxychloroquine and ivermectin, which have not been proven to be clinically efficient for COVID-19^2^ ^,^ ^3^, are widely distributed as miracle pills to constrain virus transmission at the expense of protective measures. Hydroxychloroquine may also inhibit antibody responses to vaccines and, in addition to its highly undesirable effects in the clinical-epidemiological setting of COVID-19, its indiscriminate use could impact the studies investigating immunity to this virus as well as vaccine testing^4^. In spite of this, hydroxychloroquine is still being peddled by some health professionals under pressure from politicians and government authorities.

Ivermectin is another controversial drug that is being used not only to treat but also to prevent COVID-19 in Brazil, despite lack of any confirmation of its effect on clinical outcomes^5^. The use of ivermectin (and, for that matter, antibiotics) may also hinder immunity to the severe acute respiratory syndrome coronavirus (SARS-CoV)-2 because they can modify the user's intestinal microbiota^6^ and thus impact their ability to mount effective responses to several vaccines^7^ ^,^ ^8^. It is reasonable to expect that ivermectin and antibiotics, in general, will also affect immunity to SARS-CoV-2, especially in those who employ ivermectin prophylactically, as occurs in Brazil. Therefore, the consequences of this widespread self-medication for COVID-19 could potentially contribute to the infection spreading and long-term prevalence of the epidemic in this country.

The consequences of such improper approaches for mitigating COVID-19 are worrying, leading to illusionary feelings of immunity to the virus, which underlie misguided behaviors such as not using masks and not observing social distancing by people for whom taking these medicines is the panacea for the pandemic's control. Indeed, social isolation, especially the lockdown, is a fundamental measure to control the virus spread^9^. Otherwise, the recent premature reopening of non-essential services in Brazil may further increase the rate of COVID-19 spread in the country^10^. Considering face masks, further reasons for encouraging their use are the issue recently raised by Gandhi and Rutherford regarding their potential for promoting "variolization" in individuals. This concept must be taught to the lay population in order to increase adherence^11^. Understanding the determinants by which people resist adopting protective measures is clearly of great importance so that public policies based on social isolation could have their desired effects - avoiding or reducing non-adherence to control measures.

Regarding the Health Belief Model (HBM)^12^ and COVID-19 in Brazil, low income is an important factor, which must be seen in a comprehensive way, since it is associated with the reduced quantity and quality of disseminated information, housing conditions that favor contamination, and difficulty in interrupting daily activities for economic reasons^13^, as recently raised by Silva an Arbilla (2020) in the recent discussion about the new "Human Epoch" ^14^. Furthermore, even though many are worried about the likelihood of getting COVID-19, relatively few viewed themselves as being at high risk of becoming infected with SARS-CoV-2. Although some initial controlling measures aimed to constrain the spreading of COVID-19 in Brazil^15^, the consecutive political divergences may also have contributed to a lack of compliance toward protective measures by the population. In addition, following their leaders' behaviors and refusing to wear face masks is being regarded by some citizens as a means of making their political positions known. This indicates the need to increase risk perception among the public, as it can translate into preventive actions and enhance epidemic control. Thus, interventions targeting HBM dimensions could be an alternative in an attempt to control COVID-19 dissemination in Brazil. Moreover, we should be able to identify the agnotological strategies in order to have a more efficient response against the pandemic, which should be mainly based on scientific evidences^16^. 

Finally, in view of this unique mixture of social, economic, and cultural behaviors, the main questions that remain are what is the future of COVID-19 in Brazil and when or how it will be controlled, including adherence to vaccination when it becomes available. Considering the magnitude of the spread of the disease in such a large and diverse country, the epidemic in Brazil could lead to serious consequences inside as well as outside its borders and thus impact the adequate control of the pandemic, including vaccine efficiency.
